# NLRP3-Inflammasome Inhibition with IZD334 Does Not Reduce Cardiac Damage in a Pig Model of Myocardial Infarction

**DOI:** 10.3390/biomedicines10123056

**Published:** 2022-11-28

**Authors:** Max J. M. Silvis, Evelyne J. Demkes, Leo Timmers, Fatih Arslan, Saskia C. A. de Jager, Joost P. G. Sluijter, Arend Mosterd, Dominique P. V. de Kleijn, Lena Bosch, Gerardus P. J. van Hout

**Affiliations:** 1 Department of Cardiology, University Medical Center Utrecht, 3508 GA Utrecht, The Netherlands; 2 Department of Cardiology, Radboud University Medical Center, 6500 HB Nijmegen, The Netherlands; 3 Laboratory of Experimental Cardiology, University Medical Center Utrecht, 3508 GA Utrecht, The Netherlands; 4 Circulatory Health Laboratory, UMC Utrecht Regenerative Medicine Center, University Utrecht, 3508 GA Utrecht, The Netherlands; 5 Department of Cardiology, St. Antonius Hospital, 3430 EM Nieuwegein, The Netherlands; 6 Meander Medical Center, Department of Cardiology, 3818 ES Amersfoort, The Netherlands; 7 Department of Vascular Surgery, University Medical Centre Utrecht, 3508 GA Utrecht, The Netherlands

**Keywords:** myocardial infarction, infarct size, inflammation, NLRP3-inflammasome, cardiac function

## Abstract

NLRP3-inflammasome-mediated signaling is thought to significantly contribute to the extent of myocardial damage after myocardial infarction (MI). The purpose of this study was to investigate the effects of the NLRP3-inflammasome inhibitor IZD334 on cardiac damage in a pig model of myocardial infarction. Prior to in vivo testing, in vitro, porcine peripheral blood mononuclear cells and whole blood were treated with increasing dosages of IZD334, a novel NLRP3-inflammasome inhibitor, and were stimulated with lipopolysaccharide (LPS) and adenosine triphosphate (ATP). After determination of the pharmacological profile in healthy pigs, thirty female Landrace pigs were subjected to 75 min of transluminal balloon occlusion of the LAD coronary artery and treated with placebo or IZD334 (1 mg/kg, 3 mg/kg, or 10 mg/kg once daily) in a blinded randomized fashion. In vitro, NLRP3-inflammasome stimulation showed the pronounced release of interleukin (IL)-1β that was attenuated by IZD334 (*p* < 0.001). In vivo, no differences were observed between groups in serological markers of inflammation nor myocardial IL-1β expression. After 7 days, the ejection fraction did not differ between groups, as assessed with MRI (placebo: 45.1 ± 8.7%, 1 mg/kg: 49.9 ± 6.1%, 3 mg/kg: 42.7 ± 3.8%, 10 mg/kg: 44.9 ± 6.4%, *p* = 0.26). Infarct size as a percentage of the area at risk was not reduced (placebo: 73.1 ± 3.0%, 1 mg/kg: 75.5 ± 7.3%, 3 mg/kg: 80.3 ± 3.9%, 10 mg/kg: 78.2 ± 8.0%, *p* = 0.21). In this pig MI model, we did not observe attenuation of the inflammatory response after NLRP3-inflammasome inhibition in vivo. Consecutively, no difference was observed in IS and cardiac function, while in vitro inhibition successfully reduced IL-1β release from stimulated porcine blood cells.

## 1. Introduction

Myocardial infarction (MI) occurs when a coronary artery is occluded, causing insufficient supply of oxygen and nutrients to viable myocardial tissue. Damage to the myocardium following MI promotes an inflammatory response. This reaction is thought to be responsible for expansion of the infarct and adverse cardiac remodeling [1,2].

In the past decade, multiple preclinical studies showed that the NOD-like receptor (NLR) with a pyrin domain 3 (NLRP3)-inflammasome plays a central role in this inflammatory process following experimental MI [3,4]. This intracellular protein complex is activated following a two-step process of priming and activation. The priming signal is provided by the interaction of “damage-associated molecular patterns” (DAMPS) with “pattern recognition receptors” (PRRs) [5]. These DAMPS (e.g., Heat Shock Proteins, High Mobility Group Box 1) are released after myocardial tissue injury and directly serve as ligands for PRRs (e.g., Toll-Like Receptors). This interaction subsequently leads to the upregulation of the separate NLRP3 inflammasome components via activation of nuclear translocation of various transcription factors (e.g., Nuclear Factor Kappa-light-chain-enhancer of activated B cells (NF-κB)). Following this initial priming step, the activation signal is again provided by DAMPS (e.g., extracellular ATP), leading to the assembly of the NLRP3 inflammasome [5]. This multiprotein complex consists of the innate immune receptor, NLRP3, apoptosis-associated speck-like protein containing a caspase recruitment domain (ASC), and caspase-1 [6]. The main consequence of NLRP3-inflammasome activation is the conformational change of caspase-1, enabling it to cleave the potent pro-inflammatory cytokines, interleukin (IL)-1β, and IL-18 into their active forms. In addition, caspase-1 induces a proinflammatory form of regulated cell death named pyroptosis [5].

Knock-out animal models of NLRP3-inflammasome components revealed a role for the NLRP3-inflammasome in MI when wild type mice that were subjected to MI showed significant larger infarct sizes (ISs) compared to mice that were deficient for ASC and caspase-1 [7]. These observations were further supported by studies in mice that showed upregulation of ASC and caspase-1 following MI in both circulating inflammatory cells as well as cardiomyocytes [8]. These experimental findings led to the development of NLRP3-inflammasome inhibitors that were tested in preclinical studies in small animal models of MI. These studies showed that pharmacological inhibition of the NLRP3-inflammasome reduces systemic inflammation and results in a reduction of IS and the preservation of cardiac function [9,10,11,12]. Large animal models are essential for translation of the basic mechanism of action to applicable clinical therapies, and therefore it is of pivotal importance that these previously established results are tested and confirmed in clinically relevant large animal models [13,14,15].

To this extent, we have previously investigated the effect of the small molecule inflammasome inhibitor MCC950 in a large animal model of MI and showed a beneficial cardioprotective effect with only a modest effect on systemic inflammation [13]. Further clinical translation of MCC950 was halted by Pfizer [16]. Since the NLRP3-inflammasome is a promising target in the field of cardioprotection, we believe it remains important to confirm previous results with NLRP3-inflammasome inhibitors that do have the potential for translational success. IZD334 is such a drug (IZD334; ClinicalTrials.gov Identifier: NCT04086602). In our current study, we assessed inflammasome inhibition in vitro and hypothesized that NLRP3-inflammasome inhibition with IZD334 reduces IS and preserves cardiac function after MI by reducing post-MI NLRP3-mediated inflammation [17].

## 2. Materials and Methods

Full descriptions and details of the separate methodological paragraphs can be found in the Appendix A.

### 2.1. In Vitro Stimulation

To confirm the ability of IZD334 to effectively attenuate porcine NLRP3-inflammasome signaling, we used a previously standardized study protocol [13]. In short, porcine peripheral blood mononuclear cells (PBMCs) were isolated from healthy porcine animals. Whole blood samples and isolated PBMCs were stimulated with lipopolysaccharide (LPS) (1 µg/mL, L4516, Sigma-Aldrich, St. Louis, MO, USA) and increasing concentrations of IZD334. After three h of incubation, 5 mM adenosine triphosphate (ATP) (A1852, Sigma-Aldrich, St. Louis, MO, USA) was added for 1 h to induce inflammasome activation. IL-1β release was measured in the supernatant with a Luminex Immunoasssay specific for porcine IL-1β (EPX01A-66048-901, ProcartaTM Simplex, eBioscience, ThermoFisher, San Diego, CA, USA).

### 2.2. Ex Vivo Stimulation Assay after IZD334 Infusion in Pigs

To more specifically determine in vivo target engagement, whole blood was obtained at baseline (prior to IZD334 infusion) and 60 min after in vivo IZD334 infusion (10 mg/kg). These samples were then stimulated with LPS and ATP in a similar fashion as in the previously described experiments. IL-1β release was measured in the supernatant with a Luminex Immunoassay specific for porcine IL-1β (ProcartaTM Simplex, eBioscience, San Diego, CA, USA).

### 2.3. Pharmacokinetics Study

Healthy female pigs (*n* = 3) were given intravenous (IV) treatment with IZD334 (3 mg/kg) via a 30 min infusion, and blood was collected at multiple timepoints and analyzed to determine pharmacokinetics. After a wash-out period of 3 days, the pigs received an oral dose (10 mg/kg), and plasma was again collected and analyzed.

### 2.4. In Vivo MI Study

All animal experiments were approved by the local animal welfare committee of the University Medical Center Utrecht and were executed conforming to the “Guide for the Care and Use of Laboratory Animals”. All animal experiments and analyses were performed in a blinded, randomized fashion. Thirty female pigs were subjected to closed-chest LAD coronary artery balloon occlusion for 75 min followed by 7 days of reperfusion. Fifteen minutes before reperfusion, pigs were randomly assigned to IV treatment with either placebo (40 mL PBS) or a 10 mg/kg IZD334 loading dose (dissolved in 40 mL PBS) infusion for 30 min. After the first dose of IZD334, pigs were randomly assigned to three oral treatment regimens (1 mg/kg, 3 mg/kg, and 10 mg/kg). Dosing was based on in vivo pharmacokinetic findings in healthy pigs. The control (PBS) group received an oral placebo. Oral treatment was continued for six days. All pigs underwent transthoracic and three-dimensional transesophageal echocardiography before induction of ischemia and after 7 days. Furthermore, on day 7, cardiac magnetic resonance (CMR) imaging was performed. Subsequently, the LAD was occluded at the exact same site as during infarct induction. After verification of complete vessel occlusion, Evans Blue (E2129, Sigma-Aldrich, St. Louis, MO, USA) solution was infused in the left and right coronary arteries in order to assess the area at risk (AAR). Animals were sacrificed by exsanguination under anesthesia. The heart was excised, cut into 5 slices, and incubated in 1% 2,3,5-triphenyltetrazolium chloride (TTC) (1.08380, Sigma-Aldrich, St. Louis, MO, USA) in 37 degrees 0.9% NaCl for 15 min to discriminate between infarct tissue and viable myocardium. Each slice was photographed at the basal and apical side with a ruler and analyzed with ImageJ software (NIH, Bethesda, MD, USA). The investigators were blinded to the treatment group during the experiments and the analysis of the results. Detailed methods of (dobutamine stress)-echocardiography, CMR, and infarct size measurements are described in the Appendix A.

### 2.5. Sample Size Calculation

The calculation of the number of animals needed was based on our primary outcome measurement, namely, IS, as a percentage of the ischemic AAR. We aimed to detect an absolute difference in IS of 12% of the AAR between the placebo and high-dose group, as determined in a previous study testing MCC950 [13]. Sample size calculation indicated that 12 animals were needed per group when using a standard deviation of 8.8%, an alpha of 0.05, and a power of 0.9. Expecting peri-operative mortality to be 10–20%, the inclusion of 14 animals was initially intended per group (56 animals in total). Prior to the study we decided to perform a blinded, interim analysis half-way with possible termination of the study in case of a lack of trends in primary outcome measurements (IS and left ventricular ejection fraction (EF) percent measured by CMR).

### 2.6. Serological and Histological Read-Outs

Circulating leukocyte numbers at different time points after reperfusion were measured by whole-blood analysis using an automated hematological cell-counter (Cell-Dyn Sapphire, Abbott, Santa Clara, CA, USA). Plasma samples were obtained by whole-blood centrifugation at 1850× *g* and were immediately stored at −80 °C. C-reactive protein (CRP) levels were measured using solid phase DuoSet sandwich ELISA (DY1707, R&D Systems, Minneapolis, MN, USA) according to the manufacturer’s protocol. Troponin I levels were measured using a clinical chemistry analyzer (AU5811, Beckman Coulter, Woerden, The Netherlands). Histological analysis of collagen, myocardial immune cells, neutrophils, and monocyte infiltration is described in the Appendix A.

### 2.7. Statistical Analysis

All data are expressed as mean ± SD. To assess the distribution of data, Shapiro–Wilk tests were performed. In cases of *p*-values > 0.05, differences between groups were compared using a one-way ANOVA. In cases of non-normally distributed data, Kruskal–Wallis tests were used. Leukocyte and CRP levels in the four treatment groups were analyzed using mixed models. The mixed models included the group and time point as fixed factors and a random intercept for each pig. To determine whether the time course of the parameters was different for the groups, the interaction group × time point of measurement was also taken into the model (Statistical Package for Social Sciences (SPSS) Statistics, version 25.0; IBM, New York, NY, USA).

## 3. Results

### 3.1. In Vitro Assay

**Figure 1:**
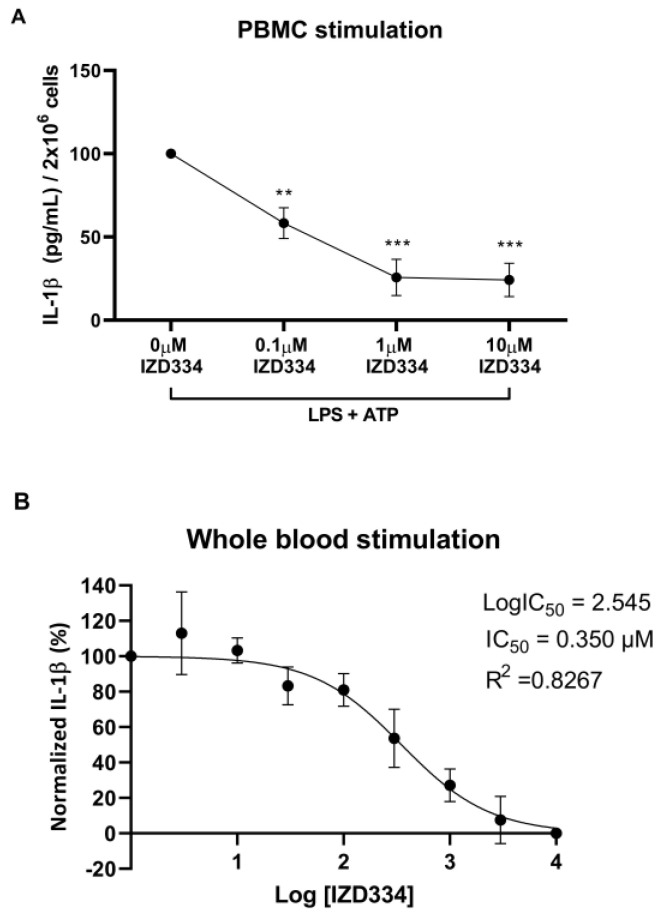
In vitro reduction of IL-1β secretion. (**A**) Porcine peripheral blood mononuclear cells (PBMCs) released IL-1β after administration of lipopolysaccharide (LPS) and adenosine triphosphate (ATP) in vitro. Addition of IZD334 reduced IL-1β release (*n* = 3). (**B**) Whole blood IC50 calculation after stimulation with LPS and ATP and administration of increasing concentrations of IZD334 in vitro. *p*-values (** <0.005, *** <0.0005) are compared to vehicle control (0 µM of IZD334).

In vitro stimulation of porcine PBMCs and whole blood with LPS and ATP led to significant IL-1β release. IZD334 dose-dependently reduced IL-1β release and reached plateau inhibition with dosing of 1 µM (*p* < 0.001)–10 µM (*p* < 0.001) (Figure 1A). In vitro stimulation and administration of IZD334 in whole blood resulted in a half maximal inhibitory concentration (IC50) of 0.350 µM (Figure 1B).

### 3.2. Ex Vivo Stimulation Assay after IZD334 Infusion in Pigs

**Figure 2:**
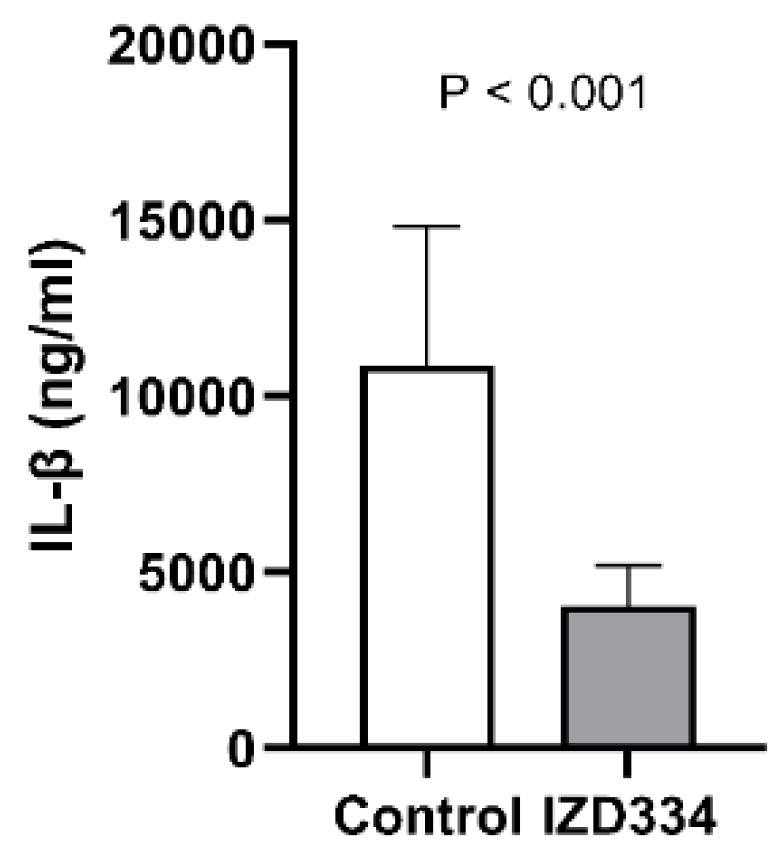
Ex vivo stimulation assay after in vivo IZD334 infusion. IL-1β release was significantly reduced when porcine whole blood was stimulated ex vivo after infusion of IZD334.

The stimulation of blood before in vivo treatment with IZD334 showed significantly higher levels of IL-1β release (11856 ng/mL ± 1969 ng/mL) compared to IL-1β release after in vivo IZD334 treatment (3968 ng/mL ± 470 ng/mL) (*p* < 0.001) (Figure 2).

### 3.3. Pharmacokinetics of IZD334

**Figure 3:**
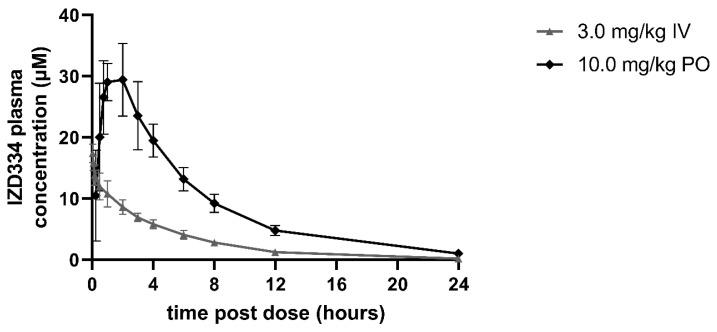
Plasma concentrations of IZD334 in healthy pigs. Concentrations following intravenous (IV) and oral (PO) administration.

In the three pigs that were used for pharmacokinetic assessment, IV administration of 3 mg/kg IZD334 led to plasma clearances (Cl) of 1.8 mL/min/kg and volume of distribution (Vdss) values of 0.58, which resulted in a calculated half-life of 3.9 h, and it maintained plasma concentrations sufficient for effective inhibition for 24 h after compound administration based on extrapolation of in vitro results. Following oral (po) administration at 10 mg/kg, absorption was rapid, with Cmax being achieved within 2 h. The bioavailability of IZD334 was 93%, and 24 h after compound administration, adequate circulating levels of IZD334 were observed (Figure 3 and Table A1), indicating measurable circulating levels of IZD334 throughout the 7-day experiment at a range compatible with in vitro NLRP3-inflammasome inhibition.

### 3.4. In Vivo Inflammasome Inhibition

#### 3.4.1. Survival and Hemodynamics

Pigs were subjected to MI and treated with either placebo or IZD334. Based on the interim analysis, which revealed similar IS in the four treatment arms, the study was aborted after inclusion of 30 pigs (63 ± 4 kg). During infarct induction and before group assignment, two pigs died due to refractory ventricular fibrillation (VF), and two pigs died one day after infarct induction (one animal in the placebo group and one animal in the low-dose (1 mg/kg) group), presumably due to late VF. This allowed a total of 26 pigs for the final analysis and a comparison of 6 pigs in the placebo group, 6 pigs in the low-dose (1 mg/kg) group, 7 pigs in the intermediate-dose (3 mg/kg) group, and 7 pigs in the high-dose (10 mg/kg) group. In one pig of the 3 mg/kg group it was not possible to measure the AAR due to a failure to infuse Evans Blue in the coronary arteries. Therefore, only in six pigs could the AAR and the IS/AAR be analyzed. Heart rate and mean arterial blood pressure were similar during the first 2.5 h after the induction of MI and subsequent compound administration (Table A2).

#### 3.4.2. Inflammatory Response after MI

**Figure 4:**
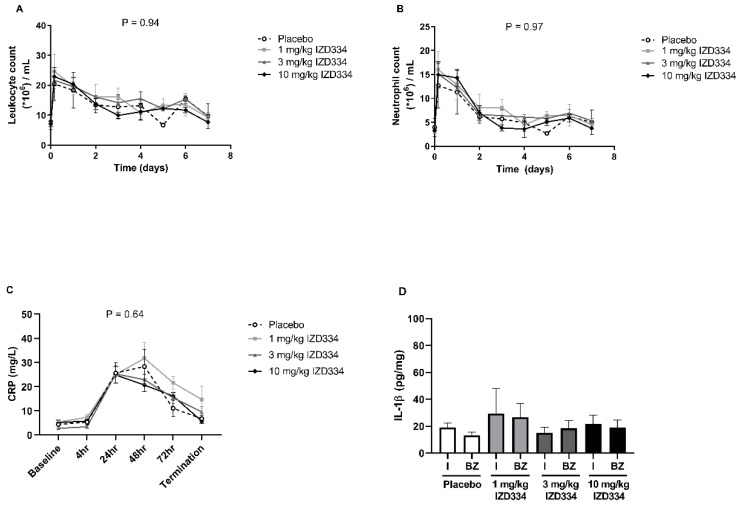
Serological and histological inflammatory read-outs. (**A**) Circulating levels of leukocytes and (**B**) neutrophils during the 7-day follow-up period. (**C**) Circulating levels of C-reactive protein (CRP) during the 7-day follow-up. (**D**) Myocardial interleukin (IL)-1β levels, measured in infarct (I) and border zone (BZ) regions of the pig heart. Data are presented as mean ± SEM (*p*-values following mixed models testing). N.B. The first three timepoints of the analysis are baseline, 4 h after reperfusion, and 24 h.

To assess the in vivo inflammatory response after MI and determine the effect of inflammasome inhibition on this response, CRP concentrations and circulating leukocyte (subset) numbers were assessed. CRP levels increased up to 24–48 h after myocardial ischemia reperfusion injury. No significant difference was seen among treatment arms at any of the time points during the follow-up period (Figure 4C). Circulating leukocyte and neutrophil counts peaked within several hours post-MI. Total leukocyte numbers and neutrophil counts did not differ between groups (Figure 4A,B). Intra-myocardial IL-1β levels were low 7 days after MI without any difference among treatment arms (Figure 4D). Circulating IL-1β levels were too low to be detected (data not shown). Histology revealed no differences between the groups in collagen formation (Figure A2A), total myocardial immune cell infiltration (Figure A2B), and neutrophil (Figure A3A) and monocyte (Figure A3B) infiltration.

#### 3.4.3. Infarct Size

**Figure 5:**
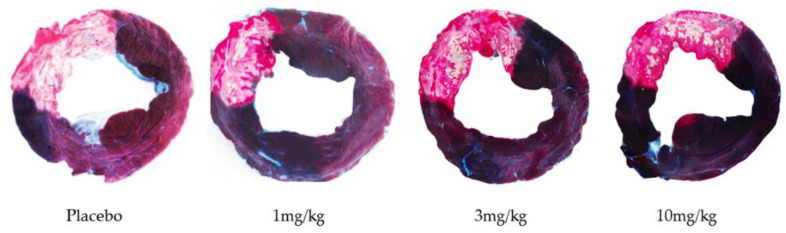
Representative pictures of basal slices of the different treatment groups (placebo, 1 mg/kg IZD334, 3 mg/kg IZD334, and 10 mg/kg IZD334. The dark area represents the remote area, the AAR is stained red, and the infarcted myocardium is stained white/pale.

**Figure 6:**
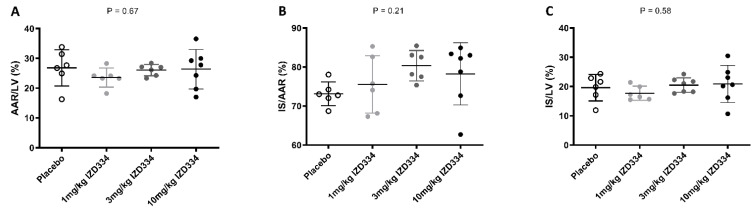
Myocardial infarct size. Normal distribution of data was confirmed as described in our methods (using Shapiro–Wilk test). Hence, a one way-ANOVA was performed. (**A**) The area at risk as a ratio of the left ventricle (AAR/LV) was similar in all treatment groups (placebo 26.8 ± 6.1%, 1 mg/kg IZD334 23.5 ± 3.2%, 3 mg/kg IZD334 26.0 ± 1.9%, 10 mg/kg IZD334 26.4 ± 6.6%, ANOVA, *p* = 0.67) groups (*p* = 0.67). (**B**) The infarct size (IS) was not different between treatment groups when expressed relative to measurements of the ischemic AAR (placebo 73.1 ± 3.0%, 1 mg/kg IZD334 75.5% ± 7.3%, 3 mg/kg IZD334 80.3 ± 3.9%, 10 mg/kg IZD334 78.2% ± 8.0%, ANOVA *p* = 0.21). (**C**) IS as percentage of the LV did not differ (placebo 19.6 ± 4.5%, 1 mg/kg IZD334 17.7 ± 6.3%, 3 mg/kg IZD334 20.5 ± 2.2%, 10 mg/kg IZD334 19.3 ± 2.9%, ANOVA *p* = 0.58). N.B. In one pig of the 3 mg/kg group, it was not possible to measure the AAR due to a failure to infuse Evans Blue in the coronary arteries. Therefore, only in 6 pigs could the AAR and the IS/AAR be shown.

**Figure 7:**
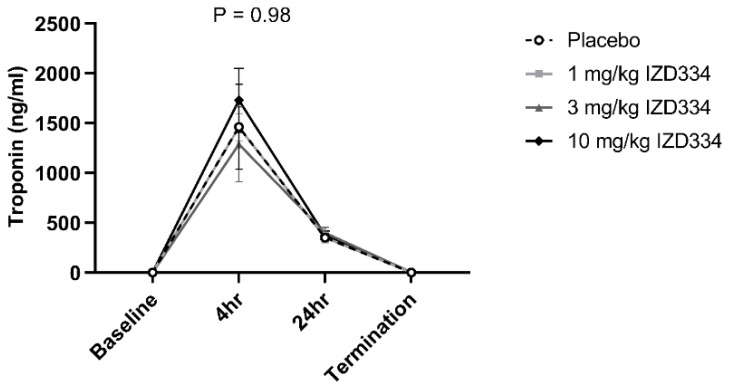
Cardiac troponin levels. Circulating levels of troponin at baseline and after reperfusion (4 h, 24 h, and prior to termination). Data are presented as mean ± SEM (*p*-values following mixed models testing).

After 7 days follow-up, IS was determined by Evans Blue TTC double staining (Figure 5). Shapiro–Wilk tests confirmed that data for all groups were normally distributed (*p*-values > 0.05 for AAR/LV, IS/AAR, and IS/LV). Hence, a one-way ANOVA was performed to assess a statistical difference between different groups. There was no difference between the AAR as a percentage of the LV (placebo 26.8 ± 6.1%, 1 mg/kg 23.5 ± 3.2%, 3 mg/kg 26.0 ± 1.9%, 10 mg/kg 26.4 ± 6.6%, *p* = 0.67). IS as a percentage of the AAR was not significantly different among the treatment groups (placebo 73.1 ± 3.0%, 1 mg/kg 75.5 ± 7.3%, 3 mg/kg 80.3 ± 3.9%, 10 mg/kg 78.2 ± 8.0%, *p* = 0.21). IS as a percentage of the LV did not differ either (placebo 19.6 ± 4.5%, 1 mg/kg 17.7 ± 6.3%, 3 mg/kg 20.5 ± 2.2%, 10 mg/kg 19.3 ± 2.9%, *p* = 0.58) (Figure 6 and Table 1). Systemic troponin I levels were measured to reflect the degree of cardiac damage. Troponin levels peaked after 4 h, and trends were similar between the treatment groups over the 7-day follow-up (Figure 7).

#### 3.4.4. Cardiac Function and Geometry

**Figure 8:**
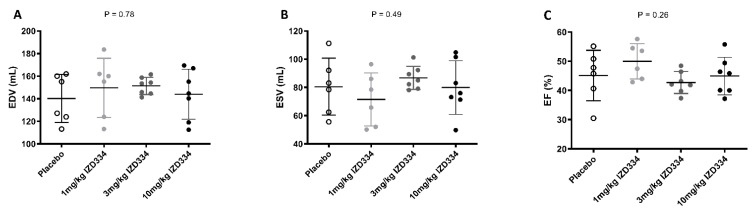
Global cardiac function measured by cardiac magnetic resonance imaging. Normal distribution of data was confirmed as described in our methods (using Shapiro–Wilk test). Hence, a one way-ANOVA was performed. No differences were observed among groups. (**A**) End diastolic volume (EDV) (placebo 140 ± 21 mL, 1 mg/kg IZD334 150 ± 26 mL, 3 mg/kg IZD334 151 ± 8 mL, 10 mg/kg IZD334 144 ± 22 mL, ANOVA *p* = 0.78). (**B**) End systolic volume (ESV) placebo 81 ± 20 mL, 1 mg/kg 72 ± 19 mL, 3 mg/kg 87 ± 8 mL, 10 mg/kg 80 ± 19 mL, ANOVA *p* = 0.49. (**C**) Ejection fraction percent (EF%) (placebo 45.1 ± 8.7%, 1 mg/kg 49.9 ± 6.1%, 3 mg/kg 42.7 ± 3.8%, 10 mg/kg 44.9 ± 6.4%, ANOVA *p* = 0.26).

Baseline cardiac function determined by echocardiography was similar in all groups (Table A4). At 7 days follow-up, pigs underwent CMR to determine cardiac volumes and function. Shapiro–Wilk tests confirmed that all data for all groups were normally distributed (*p*-values > 0.05 for end-diastolic (EDV), end-systolic ESV, and EF%). Hence, a one-way ANOVA was performed to assess the statistical difference between different groups. There was no difference in end-diastolic (EDV) and systolic (ESV) volume (EDV; placebo 140 ± 21 mL, 1 mg/kg 150 ± 26 mL, 3 mg/kg 151 ± 8 mL, 10 mg/kg 144 ± 22 mL, *p* = 0.78 and ESV; placebo 81 ± 20 mL, 1 mg/kg 72 ± 19 mL, 3 mg/kg 87 ± 8 mL, 10 mg/kg 80 ± 19 mL *p* = 0.49). Left ventricular EF was also similar in all four groups (placebo 45.1 ± 8.7%, 1 mg/kg 49.9 ± 6.1%, 3 mg/kg 42.7 ± 3.8%, 10 mg/kg 44.9 ± 6.4%, *p* = 0.26) (Figure 8 and Table 1).

#### 3.4.5. Regional Cardiac Function and Dobutamine Echocardiography

**Figure A1:**
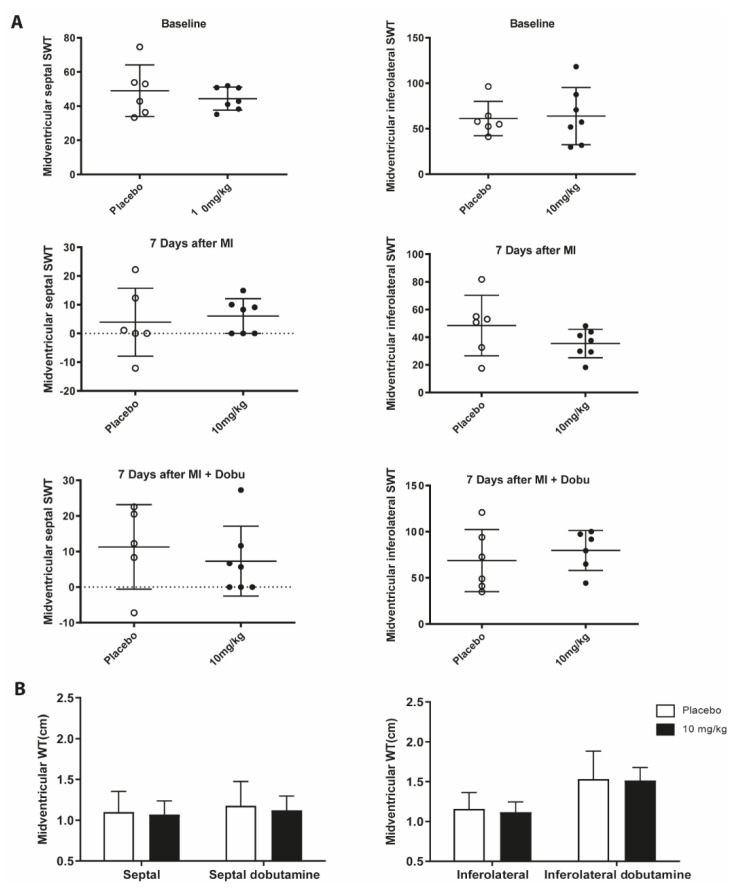
Regional cardiac function and dobutamine echocardiography in animals treated with placebo or 10 mg/kg IZD334. (**A**) Septal and inferolateral systolic wall thickening; SWT (%) at baseline and 7 days after MI with or without dobutamine. (**B**) Systolic wall thickness; WT (cm) (*n* = 5–7).

**Figure A2:**
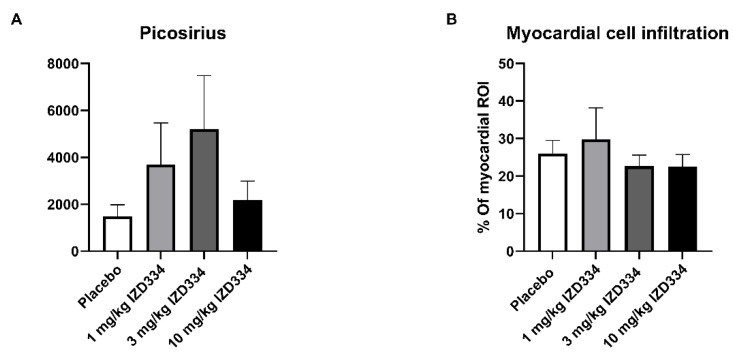
Histological analysis of infarct tissue (*n* = 6–7). (**A**) Collagen formation. (**B**) Immune cell infiltration.

**Figure A3:**
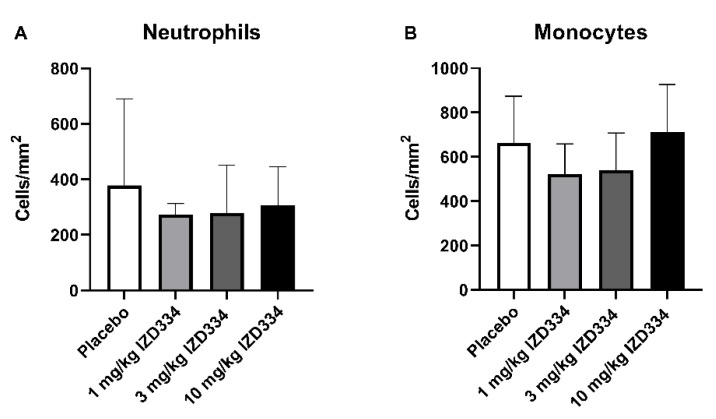
Neutrophil (**A**) and monocyte (**B**) infiltration (*n* = 6–7).

Regional cardiac function was assessed at a mid-ventricular level in the placebo and high-dose group (10 mg/kg) after 7 days follow-up. Septal and inferolateral systolic wall thickness and thickening did not differ between these groups (Figure A1). Fractional area changes at mitral, mid-ventricular, and apical levels were also equal between groups (data not shown).

#### 3.4.6. IZD334 Plasma Concentrations

**Figure 9:**
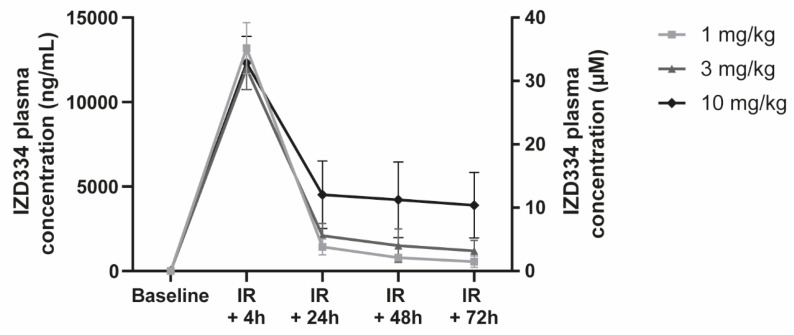
In vivo IZD334 plasma concentration measurements. Plasma measurements of IZD334 levels revealed peak concentrations 4 h (h) post-IR in all groups, consistent with an initial dosing of 10 mg/kg. Plasma levels during follow-up were consistent within each group and increased with dose across the dose range (1, 3, and 10 mg/kg IZD334).

In vivo IZD334 plasma concentrations were measured in the pigs that were subjected to MI. Concentrations were consistent across groups 4 h post-MI after the loading dose of 10 mg/kg and resulted in an average plasma concentration of 125,000 ng/mL (Figure 9 and Table A3). In the 1 mg/kg group, concentrations following oral administration were above the estimated pig whole blood IC50 for approximately 14 h post dose, albeit below the pig IC90 for the dosing duration. The concentrations in pigs treated with 3 mg/kg were above the IC50 over the 24 h dosing interval and above the estimated IC90 for approximately 8 h post dose. In the 10 mg/kg group, concentrations were above the IC50 over the dosing interval and above the estimated IC90 for approximately 15 h post dose.

## 4. Discussion

In the current study, we evaluated the systemic inflammatory response after MI in a porcine model and aimed to assess whether this response was NLRP3-inflammasome dependent. We establish that in vitro activation of the NLRP3-inflammsome profoundly occurs in a standardized porcine stimulation assay of both PBMCs and whole blood and is very effectively inhibited in vitro by IZD334. Importantly, IV and oral administration of this inhibitor results in pharmacologically active circulating concentrations, suggesting continuous inhibition of the signaling cascade. Porcine blood drawn after in vivo IZD334 infusion also showed a marked decrease in IL-1β release after ex vivo stimulation.

Despite this evidence of adequate target engagement, we did not detect an effect of NLRP3-inflammasome inhibition on the local or systemic inflammatory response in the porcine model. Accordingly, no differences in IS and left ventricular EF one week after MI in this pig model were observed. These neutral results in this large animal model are partly in contrast with previous experimental and preclinical studies of us and others that do show (systemic) anti-inflammatory consequences and consecutive cardioprotective effects of inhibitors of the NLRP3-inflammasome after MI [9,10,11,13,18].

### 4.1. Positive Effects of NLRP3-Inflammasome Inhibition in Animal Models

Since the first report on involvement of the NLRP3-inflammasome in infarct development after MI, numerous small-animal studies have evaluated its role. Previous reports showed upregulation of the NLRP3-inflammasome after MI both in mice and rat cardiac fibroblasts [7,19]. Mice deficient in ASC and caspase-1 developed smaller IS compared to wild type mice when subjected to myocardial IR injury [7]. Recently, NLRP3 deficiency in mice was shown to attenuate mortality, maladaptive remodeling, and development of HF following MI [12]. Furthermore, silencing RNA for NLRP3 or P2X7 (a receptor involved in inflammasome activation) in a mouse model of acute MI limited IS [8].

These observations were further supported by mouse studies investigating the effects of pharmacological NLRP3-inflammasome inhibition. Administration of a NLRP3-inflammasome inhibitor, derived from glyburide, showed to significantly reduce IS in a mouse model of IR injury [10]. Similar beneficial effects were seen in a mouse model of permanent left coronary artery ligation [18]. More recently, OLT1177 (Dapansutrile), a compound that inhibits the ATPase activity of NLRP3, limited infarct development and prevented left ventricular dysfunction following IR injury in mice [11].

### 4.2. Neutral and Negative Effects in Animal Models

Importantly, neutral and negative effects of NLRP3-inflammasome inhibition in the setting of experimental myocardial IR injury were also observed [20,21]. Mice deficient for ASC did not show a reduction of IS in a model of MI, and NLRP3-deficient mice showed larger infarcts compared to wild type mice [20]. A possible explanation for the heterogeneity between these studies may be the double edged sword inflammation plays in infarct development after MI [22,23]. NLRP3-inflammasome activity could possibly play multiple conflicting roles, comparable to other inflammatory mediators, such as TNF-α, high-mobility group box 1, and toll-like receptor 2 [21,24]. Additionally, evidence of NLRP3-inflammasome activation following MI in humans is scarce and limited to a few autopsy cases [7].

Previously, our group showed that treatment with a small molecule inhibitor, MCC950, dose-dependently reduced IS accompanied by an improvement in LV function at 7 days following MI in a large animal model. However, circulating markers of inflammation were only modestly lower in the pigs that were treated with MCC950, while a clinically relevant effect on cardiac function was observed. Interestingly, differences in myocardial IL-1β levels did not reach statistical significance in our previous study. One could hypothesize that the difference in inflammatory markers observed in our previous study could be an effect rather than a cause of the attenuated cardiac damage. This could possibly suggest pleiotropic effects of MCC950 and the absence of such pleiotropic effects of IZD334, although evidence for this hypothesis is currently lacking [13].

Myocardial and circulating IL-1β levels were very low in the current study, possibly indicating modest NLRP3-inflammasome activity 7 days after MI. This is in contrast with our previous study. The assay used in our previous study was currently not available, and standardization of IL-β measurements remains very challenging, with considerable variability between measurements [25]. The lack of reduced circulating CRP and leukocyte quantities suggests that the inflammatory reaction that is observed in our model is either caused by signaling pathways other than the NLRP3-inflammasome, or alternative rescue cascades neutralize successful inhibition.

The latter is theoretically possible since different inflammasomes have been characterized and at least account for some IL-1β activity in a normal situation [26]. In a diseased state, when the NLRP3-inflammasome is inhibited, it could be that these other inflammasomes take over the role of the NLRP3-inflammasome, resulting in a similar net effect.

Given the effectiveness of the NLRP3-inflammasome inhibitor tested in vitro and the adequate measured in vivo levels of the compound, we therefore hypothesize that in the current model of MI, NLRP3-inflammasome activation was either not present, or if present was not essential for systemic inflammation and final IS and cardiac function after MI.

### 4.3. Mechanism of Action and Target Engagement

There is much similarity regarding the chemical structure of MCC950 and IZD334. The binding-site and mechanism-of-action of MCC950 have previously been published [27]. MCC950 inhibits ATP hydrolysis and therefore activation of the NLRP3 molecule by binding to the Walker-B motif, and IZD334 is believed to act in a similar way [27]. IZD334 has been patented, and the effectivity of the compound has been evaluated in standardized human assays of pyroptosis and IL-1β- release directly against MCC950 [28]. These results are comparable to our whole blood assay in which IZD334 performs as well as MCC950. IZD334, like MCC950, is believed to prevent inflammasome assembly and not protein formation, thus acting through posttranslational regulation of these proteins. Hence, mRNA levels and even protein levels of these different inflammasome components (NLRP3, ASC, Caspase-1) do not always reflect its actual activity. Translational studies with NLRP3-inflammasome assembly inhibitors have to overcome these challenges. Many studies that were recently performed with murine knock out models and, e.g., mRNA silencers use read-outs for NLRP3-inflammasome activity that are mechanistically not altered by inhibition of inflammasome assembly and therefore not usable as read-outs for target engagement in our study.

### 4.4. Translational Failure

Given the heterogeneity of animal MI models and study design, it is imaginable that the role of the NLRP3-inflammasome in these models differ, possibly explaining the contradictory results observed when inhibiting this mechanism. The follow-up in our study is longer than in most small-animal studies, possibly indicating that the effect is time-dependent [9,11]. In our model, we can therefore not rule out that any beneficial effects of inhibition in the early phase after MI were diminished after our 7-day follow-up period. Furthermore, involvement of surgical injury in NLRP3-inflammasome activation in MI models was suggested. De Jong et al. showed that NLRP3-activity was only detected in the hearts of the open chest mouse model, while it was not present in a closed-chest model [29].

In translational cardiology, a decreased effect of cardioprotective therapies when moving along the translational axis from small to large animals is recognized [15,30]. Introducing comorbidities; risk factors including hypertension, hyperlipidemia, and diabetes; and co-medications in animal models could improve the chance of translational success. The failure to reproduce beneficial effects from previous preclinical studies in small and large animal models therefore again underlines the importance of further research and repeated testing with different NLRP3-inflammasome inhibitors, dosages and routes of administration in clinically relevant large animal models before heading to the clinical MI setting.

### 4.5. Clinical Implications

In the current study we do not provide evidence that NLRP3-inflammasome inhibition is beneficial in the acute setting after myocardial infarction. We therefore believe that further clinical translation in this specific disease should not be pursued. In the setting of atherosclerosis and the prevention of major cardiovascular events, anti-inflammatory strategies have recently been shown to be beneficial [31,32,33,34]. These results could indicate a role for NLRP3-inflammasome inhibition in preventive cardiovascular medicine.

## 5. Conclusions

In conclusion, our study does not provide evidence that the inflammatory reaction that occurs after MI is solely dependent on the NLRP3-inflammasome, since continuous inhibition of the NLRP3-inflammasome-mediated signaling using IZD334 over a 7-day follow-up period does not decrease the inflammatory response in a pig model of MI. Consequently, we did not observe functional, serological, or histological evidence of decreased cardiac damage.

## Figures and Tables

**Table 1:** Infarct size and cardiac function 7 days after MI and treatment. AAR, area at risk; LV, left ventricle; IS, infarct size. EDV, end-diastolic volume; ESV, end-systolic volume; EF, ejection fraction.

Group	AAR/LV ± SD	IS/AAR ± SD	IS/LV ± SD	EDV (mL)	ESV (mL)	EF%
Placebo (*n* = 6)	26.8 ± 6.1%	73.1 ± 3.0%	19.6 ± 4.5%	140.2 ± 21.2	80.5 ± 20.2	45.1 ± 8.7%
1 mg/kg IZD334 (*n* = 6)	23.5 ± 3.2%	75.5 ± 7.3%	17.7 ± 6.3%	149.6 ± 26.2	71.5 ± 18.7	49.9 ± 6.1%
3 mg/kg IZD334 (*n* = 7)	26.0 ± 1.9%	80.3 ± 3.9%	20.5 ± 2.2%	151.4 ± 7.6	86.8 ± 8.2	42.7 ± 3.8%
10 mg/kg IZD334 (*n* = 7)	26.38 ± 6.6	78.2 ± 8.0%	19.3 ± 2.9%	144.0 ± 22.0	80.0 ± 18.9	44.9 ± 6.4%

## Data Availability

Data supporting the findings of this study are available from the corresponding author upon reasonable request.

## Appendix A

### Materials and Methods

1. Animals

A total of 33 female Landrace pigs (63 ± 4 kg) was evaluated in this study (Van Beek, Lelystad, The Netherlands). Three pigs were used for pharmacokinetic evaluation. Thirty female pigs were subjected to MI by 75 min of transluminal balloon occlusion of the mid left anterior descending artery (LAD). All animals were conventionally housed in stables with concrete floors and hay bedding with a light/dark cycle of 12/12 h and water ad libitum. Rubber bite sticks were provided as environmental enrichment.

2. Circulating levels of IZD334

In vivo IZD334 concentrations were measured in plasma samples using mass-spectrometry. IZD334 content was quantified using positive ionization mode and multiple reaction monitoring on a Sciex API 5500 Mass spectrometer (MRM transition: 392.0 > 176.1).

3. In vitro stimulation assay

In short, porcine peripheral blood mononuclear cells (PBMCs) were isolated from healthy porcine animals using Ficoll density-gradient centrifugation. Whole blood samples and isolated PBMCs were stimulated with lipopolysaccharide (LPS) (1 µg/mL, L4516, Sigma-Aldrich, St. Louis, MO, USA) and increasing concentrations of IZD334 (top concentration of 10 µM with an 8-point half-log dilution). After three h of incubation, 5 mM adenosine triphosphate (ATP) (A1852, Sigma-Aldrich, St. Louis, MO, USA) was added for 1 h to induce inflammasome activation. IL-1β release was measured in the supernatant with a Luminex Immunoasssay specific for porcine IL-1β (ProcartaTM Simplex, eBioscience, San Diego, CA, USA).

4. Pharmacokinetics

At multiple time points (0.033, 0.083, 0.17, 0.25, 0.50, 1.0, 2.0, 3.0, 4.0, 6.0, 8.0, 12.0, 24.0 h) after dosing, plasma was collected by whole-blood centrifugation at 1850× *g*, and samples were immediately stored to determine pharmacokinetics. After a wash-out period of 3 days, the pigs received an oral dose (10 mg/kg), and plasma was collected again at multiple time points (0.25, 0.50, 0.75, 1.0, 2.0, 3.0, 4.0, 6.0, 8.0, 12.0, 24.0 h) for pharmacokinetic purposes. Samples were sent to Sygnature Discovery (Nottingham, United Kingdom) to determine circulating levels of IZD334.

The volume of distribution at steady state (Vdss) is defined as the total concentration of IZD334 in the entire body divided by IZD334 plasma concentration. Drug clearance (Cl) was calculated by dividing the total administered dose with the area under the curve. Maximal concentration (Cmax) was obtained by calculating the mean of the highest concentrations of the separate pigs. Bioavailability (F%) was calculated using the following equation: (100 ∗ ((area under the curve oral dose × IV dose)/(area under the curve IV dose × oral dose))).

5. Oral premedication, anesthesia, and analgesia in vivo study

The preoperative protocol was as previously described in detail [13]. Pigs were pre-treated with amiodarone (14860864, Mylan, Bunschoten, The Netherlands) for 10 days (1200 mg loading dose, 800 mg/day maintenance); clopidogrel (B01AC04, Mylan, Bunschoten, The Netherlands), 75 mg/day; and acetylsalicylic acid (B01AC06, Teva, Haarlem, The Netherlands) (loading dose 7 days before the experiment, 80 mg/day maintenance). One day before surgery, animals received a buprenorphine (N02AE01, Grunentahl, Breukelen, The Netherlands) patch (5 µg/h). At the day of surgery, anesthesia and analgesia was induced by intramuscular injection of ketamine (QN01AX03, Vetoquinol, Breda, The Netherlands) (15 mg/kg), midazolam (N05CD08, Aurobindo, Baarn, The Netherlands) (0.75 mg/kg), and atropine (A03BA01, Centrafarm, Etten-Leur) (0.015 mg/kg) followed by IV administration of thiopenthal (N01AF03, Eureco-Pharma, Ridderkerk, The Netherlands) (4 mg/kg). Pigs were intubated and connected to a respirator with a 1:2 oxygen and air ratio. Continuous sedation was achieved with IV pancuronium (S2497, Selleckchem, PA, USA) (0.1 mg/kg/h), midazolam (0.4 mg/kg/h), and sufentanil (N01AH03, Pharma GMBH, Hameln, Germany) (2.5 µg/kg/h).

6. Echocardiography

Images were made using a X7-2T transducer on an iE33 ultrasound device (Philips, Eindhoven, The Netherlands). The pig was placed in the right lateral position, and the echo probe was inserted 50–60 cm into the esophagus. We used the 3D full volume option and temporarily interrupted mechanical ventilation to assess LV geometry and function. On day 7 we additionally obtained dobutamine stress-echocardiography to assess regional cardiac function. Images were analyzed in a blinded fashion with validated software, QLab 10.7 (Philips, Eindhoven, The Netherlands).

7. Cardiac Magnetic Resonance Imaging

Animals were placed on the CMR table in a supine position, under continuous sedation as described above. Steady-state free precession (SSFP) short axis images from the left ventricle (LV) base through the LV apex were used for the quantification of LV volumes, ejection fraction (EF), and mass. Imaging was performed using a respiratory-corrected, cardiac-gated SSFP in sequence. CMR images were anonymized and analyzed offline using MedisSuite (Medis Medical Imaging Systems, version 3.1, Leiden, The Netherlands).

Infarcted and border zoned myocardial tissues were collected, and protein was isolated using a Roche protein isolation kit (cOmplete™ Lysis-M EDTA-free, Roche, Basel, Switzerland). IL-1β protein was measured with a Luminex Immunoassay specific for pig IL-1β (ProcartaPlex Pig IL-1β simplex Cat# EXP01A-66048-901 Invitrogen, Thermo Fisher Scientific, MA, USA) and corrected for total protein. For further histological analysis, infarcted and BZ myocardial tissue was processed, paraffin-imbedded, and cut into 5 µm sections after being conserved in 4% paraformaldehyde for 7 days. Cell nuclei were stained using hematoxylin, and area of cell infiltration was counted for each pig in 5 regions close to fibrotic areas in the infarct zone using Olympus CellSens software. Collagen was visualized using Picosirus Red staining (Abcam). The percentage of collagen was quantified using ImageJ software (1.47v).

Neutrophil infiltration in myocardial tissue was quantified in paraffin-embedded histological biopsies that were conserved in 4% formaldehyde for at least 7 days with a monoclonal mouse antibody against porcine neutrophils (Clone PM1, BMA Biomedicals, Augst, Switzerland) for 60 min and secondary incubation with Brightvision Poly-AP-anti-mouse (ImmunoLogic, Duiven, The Netherlands) for 30 min followed by development with liquid permanent red.

Monocyte infiltration was quantified in histological slides of myocardial tissue (paraffin and fresh frozen slides, respectively) using a monoclonal mouse anti-pig antibody against CD107a (Clone JM2E5, Bio-Rad, Kidlington, UK) or a mouse monoclonal antibody against CD14 (Ab23919, Abcam, Cambridge, UK); 60 min of incubation was followed by Brightvision Poly-AP-anti-mouse (ImmunoLogic, Duiven, The Netherlands) for 30 min and development with liquid permanent red.

**Table A1:** Histological analysis and myocardial IL-1β measurement.

IZD334		
Route	PO	IV
T_1/2_ (h)	-	3.9
MRT_0-inf_ (h)	-	5.3
Cl (mL/min/kg)	-	1.8
Vd_ss_ (L/kg)	-	0.58
C_max_ (ng/mL)	12,116	-
AUC_0-inf_ (ng.h/mL)	86,337	27,786
F%	93	

**Table A2:** Pharmacokinetic parameters and intravenous (IV) and oral (PO) concentration time profiles of IZD334 in pigs (*n* = 3). Abbreviations: T1/2, half-life; MRT0-inf, mean residence time from time 0 extrapolated to infinite time; Cl, drug clearance; Vdss, steady-state method; Cmax, maximal concentration; AUC0-inf, area under the curve from time 0 extrapolated to infinite time; F, bioavailability.

Treatment Group	0 Min	30 Min	60 Min	90 Min	120 Min	150 Min
	HR (bpm)	MAP (mmHg)	HR (bpm)	MAP (mmHg)	HR (bpm)	MAP (mmHg)
Placebo (*n* = 6)	74 ± 6	71 ± 15	67 ± 8	60 ± 16	67 ± 10	72 ± 20
1 mg/kg IZD334 (*n* = 6)	70 ± 12	85 ± 7	68 ± 9	71 ± 12	67 ± 8	79 ± 11
3 mg/kg IZD334 (*n* = 7)	74 ± 4	74 ± 13	72 ± 11	58 ± 20	61 ± 7	70 ± 23
10 mg/kg IZD334 (*n* = 7)	73 ± 11	72 ± 12	70 ± 11	56 ± 19	62 ± 7	62 ± 15

HR = heart rate, bpm = beats per minute, MAP = mean arterial blood pressure, mmHg = millimeters mercury.

**Table A3:** Plasma levels of IZD334 in vivo.

Group	Timepoint	Concentration (ng/mL)		
		Mean		SD
1 mg/kg IZD334				
(*n* = 6)	Reperfusion + 4 h	13,192	±	1508
	Day 1	1434	±	482
	Day 2	795	±	165
	Day 3	555	±	337
	Day 7	44	±	9
3 mg/kg IZD334				
(*n* = 7)	Reperfusion + 4 h	11,992	±	1256
	Day 1	2090	±	724
	Day 2	1501	±	989
	Day 3	1190	±	626
	Day 7	120	±	32
10 mg/kg IZD334				
(*n* = 7)	Reperfusion + 4	12,312	±	1585
	Day 1	4522	±	2005
	Day 2	4213	±	2248
	Day 3	3887	±	1948
	Day 7	233	±	84

**Table A4:** Left ventricular volumes and ejection fraction (EF) measured with echocardiography at baseline. Abbreviations: EDV, end-diastolic volume; ESV, end systolic volume; EF, ejection fraction.

Group	EDV (mL)	ESV (mL)	EF%
Placebo (*n* = 6)	106 ± 9.5	43.2 ± 3.3	59.1 ± 1.8%
1 mg/kg IZD334 (*n* = 6)	112 ± 13.0	47.7 ± 7.0	57.5 ± 1.7%
3 mg/kg IZD334 (*n* = 7)	114.6 ± 13.6	47.4 ± 7.0	58.7 ± 1.6%
10 mg/kg IZD334 (*n* = 7)	117 ± 12.1	47.8 ± 5.1	59.2 ± 1.0%
